# Caracterización de genomas recombinantes de la variante ómicron del SARS-CoV-2 entre el 2022 y el 2023 en el departamento de Antioquia, Colombia

**DOI:** 10.7705/biomedica.7698

**Published:** 2026-06-01

**Authors:** Cristian Arbey Velarde, Leila Cristina Vega, Idabely Betancur

**Affiliations:** 1 Laboratorio Departamental de Salud Pública de Antioquia, Secretaría Seccional de Salud y Protección Social de Antioquia, Gobernación de Antioquia, Medellín, Colombia Gobernación de Antioquia Medellín Colombia

**Keywords:** recombinación genética, genoma viral, SARS-CoV-2, filogenia, Recombination, genetic, genome, viral, SARS-CoV-2, phylogeny

## Abstract

**Introducción.:**

La recombinación es un mecanismo que evita la acumulación de mutaciones nocivas. Para que se dé entre diferentes sublinajes de un mismo virus, se requiere cocirculación y coinfección en un mismo huésped. Mediante NGS *(next-generation sequencing)* se han podido caracterizar linajes recombinantes de la variante ómicron.

**Objetivo.:**

Describir cómo la recombinación viral en linajes de ómicron le ha conferido características importantes para su prevalencia sobre otros linajes.

**Materiales y métodos.:**

Se caracterizaron 338 genomas de linajes recombinantes de muestras positivas de SARS-CoV-2 analizadas por el Laboratorio Departamental de Salud Pública de Antioquia, entre el 9 de noviembre del 2022 y el 5 de noviembre del 2023. Los genomas fueron obtenidos mediante la tecnología de secuenciación de Oxford Nanopore™. Por medio de análisis filogenéticos se identificaron diferentes sublinajes recombinantes de ómicron. Predictores permitieron la identificación de regiones del genoma de SARS-CoV-2 que habían sido producto de eventos de recombinación.

**Resultados.:**

Se identificaron 26 linajes recombinantes de ómicron en los que se encontraron cambios de aminoácidos de gran importancia, involucrados en la evasión de la respuesta inmunitaria, infectividad y replicación viral. También se identificó la mutación F456L, presente en el 55 % de los genomas de XBB.1.5.72, que sirve como biomarcador de reconocimiento de este sublinaje. Los análisis con predictores permitieron la identificación de los probables linajes parentales de algunos genomas recombinantes como XBB.1.5 y XBB.1.5.77.

**Conclusión.:**

La circulación de sublinajes recombinantes de ómicron muestran cambios importantes que impactan la biología de estos y han alterado los factores de infección y virulencia.

## Abstract (en)

**Introduction.:**

Recombination is a mechanism that prevents the accumulation of noxious mutations. Recombination occurs between different sublineages of the same virus that are co-circulating and co-infecting the same host. Next-generation sequencing technologies have allowed the identification of recombinant sublineages of omicron.

**Objective.:**

To describe how viral recombination in new omicron lineages has conferred important characteristics to this variant and allowed its prevalence.

**Materials and methods.:**

A total of 338 genomes of recombinant lineages from positive samples of SARS-CoV-2 analyzed by the *Laboratorio Departamental de Salud Pública de Antioquia* were characterized, between November 9, 2022, and November 5, 2023. The genomes were obtained using Oxford Nanopore™ sequencing technology. Phylogenetic analysis identified different recombinant sublineages of omicron and predictors allowed the identification of regions in the SARS-CoV-2 genome that were the product of recombination events.

**Results.:**

Twenty-six recombinant lineages of omicron were identified, and important amino acid changes were found involved in evasion of the immune response, infectivity and increased viral replication, N501Y, D614G and P681H. The F456L variant was also identified and was present in 55% of the XBB.1.5.72 genomes and is a biomarker for recognition of this sublineage. The analysis with recombination predictors allowed the identification of the potential parents of some recombinant genomes such as XBB.1.5 and XBB.1.5.77.

**Conclusion.:**

The circulation of omicron sublineage recombinants shows important changes that impact their biology and have altered infection and virulence factors.

Las mutaciones del material genético son parte del proceso evolutivo de todos los organismos. Tanto los microorganismos que tienen por material genético el ADN, como los virus ARN, presentan fenómenos de recombinación. Entre los virus, los ARN tienen la tasa de mutación más elevada; sin embargo, algunos virus tienen en su maquinaria genética un sistema de autocorrección, por lo que mutan más lentamente; este es el caso del SARS-CoV-2. Es importante mencionar que algunas de las mutaciones pueden ser nocivas para la replicación y, por tanto, para la supervivencia del virus, mientras que otras pueden ser neutras y otras pueden conferirle ventajas selectivas ^1^^-^^3^.

Existen varios tipos de recombinación, entre los que se encuentra la recombinación clásica y la no clásica. La recombinación clásica implica la ruptura de los enlaces covalentes dentro del ácido nucleico y el intercambio de información genética. Este tipo de recombinación por ruptura y reparación no es común en los virus de ARN; en este tipo de virus, la recombinación contempla mecanismos más complejos ^4^.

La recombinación no clásica, en cambio, implica el rearreglo o reordenamiento del material genético, que puede provenir de dos viriones diferentes. Las recombinaciones se pueden dar por intercambio de material genético, lo cual resulta en la formación de un ácido nucleico compuesto de dos o más ácidos nucleicos parentales; además, pueden ocurrir entre genomas virales diferentes o entre un genoma viral y otro no viral ^4^. Para que se den eventos de recombinación entre diferentes linajes de un mismo virus, por ejemplo, en el SARS-CoV-2, se requiere que se dé una coinfección con más de una variante del virus en el mismo huésped ^5^.

Desde la caracterización genómica inicial del SARS-CoV-2, se ha identificado que los determinantes clave en la velocidad con la que evoluciona este virus y en su diversificación en los diferentes clados genéticos son su alta tasa de mutacional y los procesos repetitivos de recombinación característicos de los betacoronavirus ^6^^-^^9^.

En las Américas, la variante ómicron se detectó por primera vez a finales de noviembre del 2021 y se propagó rápidamente hasta convertirse en el clado predominante en toda la región. Al 12 de abril de 2022, ómicron se había convertido en la única variante notificada en las muestras positivas de 53 países, detectándose en el 100 % de las muestras secuenciadas ^6^. Esta variante demostró una alta transmisibilidad, fuertemente correlacionada con la identificación de mutaciones puntuales que, además, permitieron caracterizar diferentes sublinajes dentro de la misma variante.

Hasta el 12 de abril del 2022, se habían reportado a nivel global cinco diferentes sublinajes de ómicron: BA.1, BA.2, BA.3, BA.4 y BA.5., y la BA.2 era la más predominante en la mayoría de las regiones.

En el continente americano, los sublinajes BA.1 y BA.1.1 se convirtieron en predominantes, y se identificaron en más del 97 % de las muestras caracterizadas desde la introducción de ómicron en América ^6^.

En Antioquia, entre el 2022 y el 2023, se presentó la circulación conjunta a nivel local de linajes y sublinajes de las variantes delta y ómicron. Para ese momento, se identificaron algunos casos de coinfección por ambas variantes, que llevaron a eventos de recombinación en varios pacientes infectados. A través de la técnica de secuenciación genómica de nueva generación *(nextgeneration sequencing,* NGS), se lograron identificar nuevos linajes y sublinajes producidos por dichos eventos.

El sistema de nomenclatura Pangolin nombró a estos sublinajes productos de la recombinación con el prefijo X seguido de diferentes sufijos en función del tipo de sublinajes identificado ^10^. Es así como en Antioquia se identificó el primer linaje de SARS-CoV-2 recombinante, llamado XBB.1, el 9 de noviembre del 2022.

En la mayoría de los casos, los genomas recombinantes de ómicron surgieron a partir de la recombinación genética entre sublinajes de esta misma variante, tales como BQ.1.1, BQ.1.1.4 y BE.1.1.1, que, aunque no representaban más del 10 % de los genomas secuenciados entre enero del 2022 y diciembre del 2023, eran los sublinajes más prevalentes y, por tanto, los que tenían mayores probabilidades de dar origen a genomas recombinantes como XBB.1 y XBB.1.5, los cuales fueron los primeros genomas de su tipo en ser identificados en el departamento de Antioquia ^11^^-^^13^.

La caracterización de los sublinajes recombinantes de ómicron se hizo por medio de la estrategia de vigilancia genómica llevada a cabo por la Red Nacional de Laboratorio para la Vigilancia Genómica COVID-19 dirigida por el Instituto Nacional de Salud. Esta red de 14 laboratorios a nivel nacional identificó, mediante secuenciación 660 genomas recombinantes de ómicron, SARS-CoV2 en Colombia, de los cuales 338 eran de Antioquia, mediante el uso de las plataformas de secuenciación Illumina™ y Oxford Nanoporos™. Estas tecnologías de secuenciación han demostrado ser una herramienta que proporciona información en tiempo real sobre las variantes circulantes del SARS-CoV-2 y, además, han permitido comprender la diversidad genómica de este virus, además de sus patrones de dispersión, mecanismos de transmisión y patogenicidad ^14^. Esta estrategia ha permitido identificar las diferentes variantes de preocupación, de interés y de alta importancia, que circulan en el departamento ^14^^,^^15^.

Este fue un estudio descriptivo que buscaba evaluar la diversidad genómica de los sublinajes recombinantes de ómicron y describir el impacto epidemiológico de esta variante. Este estudio cobra importancia al caracterizar la diversidad de los sublinajes de este tipo, que circularon en Antioquia entre el 2022 y 2023, mediante el análisis de los datos de genomas obtenidos a partir de tecnologías de secuenciación de próxima generación.

## Materiales y métodos

### Obtención de muestras y metadatos

Se analizaron 338 genomas recombinantes (linajes XAG, XAM, XBB.1 y derivados, y XBB.2 y derivados) con los respectivos datos sociodemográficos de la población de estudio. Estos datos corresponden a pacientes con muestras positivas de SARS-CoV-2 secuenciadas en el Laboratorio Departamental de Salud Pública de Antioquia y hacen parte de muestreos rutinarios y probabilísticos definidos por el Instituto Nacional de Salud de Colombia. Las muestras fueron tomadas en el periodo epidemiológico del 9 de noviembre de 2022 al 5 de noviembre del 2023. Los genomas analizados en este estudio se encuentran consignados en el repositorio GISAID *(Global Initiative on Sharing All Influenza Data)*^16^.

### Secuenciación de genomas mediante la plataforma de Oxford Nanopore™

Para la secuenciación del material genético obtenido de las muestras positivas identificadas mediante RT-PCR, se utilizó la plataforma de secuenciación MinION™ de Oxford Nanopore Technologies™. La preparación de la librería se hizo con el kit SQK-LSK109, empleando como identificador molecular de cada muestra el kit de código de barras EXP-NBD196, siguiendo el protocolo ARTIC network ^17^. La librería fue cargada en una Flow Cell R9.4.1, en un secuenciador MinION™ Mk1C ^18^. Para este protocolo, se empleó la versión 4.1 del esquema de cebadores *(primers)* de SARS-CoV-2.

### Análisis descriptivo

Se realizó un análisis descriptivo para determinar la proporción de linajes recombinantes de ómicron en Antioquia entre el 9 de noviembre del 2022 y el 5 de noviembre del 2023. Las muestras con datos incompletos no se incluyeron en el análisis, ni tampoco los genomas que se clasificaron como *«unassigned».* Los análisis descriptivos se llevaron a cabo en el programa RStudio™, versión 3.6.0, con los paquetes ggplot2 3.3.6 y ggpubr 0.4.0 ^19^.

### Análisis filogenético

Para construir la relación genética de los linajes recombinantes X* de ómicron, se analizaron 338 genomas obtenidos de la base de datos GISAID. Para dar confianza a la topología del árbol, se agregaron 170 genomas de los principales clados de SARS-CoV-2, tales como gamma (37 genomas), mi (40 genomas), alfa (20 genomas), delta (36 genomas) y ómicron (37 genomas no recombinantes) que cumplieron con los criterios de profundidad y cobertura (150X y 95 %, respectivamente). También se agregó un grupo externo como genoma de referencia, Wuhan (NC_045512). Todas las secuencias fueron alineadas al genoma de referencia utilizando el programa Mafft, versión 7.310; posteriormente, se editaron con Trimal, versión 1.4.15, y, por último, se generó un árbol con el programa IQTree, versión 2.0.3 ^19^^-^^21^.

Se utilizó el modelo de sustitución TIM2 + F + I y se evaluó la confiabilidad de la topología del árbol filogenético utilizando como medidas de soporte *ultrafast booptrap approximation* (-bb) con 10 000 réplicas y *approximate likelihood ratio test* (-alrt) con 1000 réplicas. La visualización y edición del árbol se realizó con el *software* FigTree, versión 1.4.3 ^22^.

### Análisis de recombinación genética

Los puntos de recombinación, también llamados *breakpoints,* en los genomas de linajes recombinantes de ómicron (XBB.1.5, XBB.1.5.72, XBB.1.5.77), fueron identificados mediante el gestor de programas de detección de eventos de recombinación, *Recombination Detection Program* (RDP), versión 4.101 ^23^. El RDP comprende una amplia gama de métodos para detectar y visualizar puntos de recombinación en los genomas virales. Este programa genera alineamientos de múltiples genomas virales e identifica los eventos de recombinación genética por cada genoma, cuáles son las posiciones más probables en el genoma donde ocurren dichos eventos y qué genomas son potenciales linajes parentales de los linajes o sublinajes recombinantes.

El repositorio de los métodos de detección de recombinación genética que está integrado en el RDP analiza cada base nucleotídica por cada trío de secuencias seleccionadas al azar; posteriormente, se infiere cuál de estos genomas es el recombinante y cuáles son sus genomas parentales. Los programas utilizados por RDP para la identificación de puntos de recombinación, además del método original RDP, fueron BootScan ^24^, MaxChi ^25^, Chimaera ^26^, 3Seq ^27^, Geneconv ^28^, LARD ^29^ y SiScan ^30^. Cada uno de estos métodos da una p, la cual indica que una región de un genoma es producto de un evento de recombinación si su valor es menor de 0,05.

Para este análisis se seleccionaron cinco genomas de cada uno de los sublinajes de ómicron, recombinantes y no recombinantes; para el primer grupo solo se seleccionaron los tres más prevalentes. Se seleccionó un total de 85 genomas de linajes, entre estos: B.1, B.1.111, B.1.621, B.1.625, BA.1.1, BA.2, BA.2.12.1, BA.4.1, BA.5.1, BQ.1.1, BQ.1.1.10, BQ.1.1.18, BQ.1.1.4, P1, XBB.1.5, XBB.1.5.72 y XBB.1.5.77.

## Resultados

### Prevalencia de linajes recombinantes entre el 9 de noviembre del 2022 y el 5 de noviembre del 2023

**Figura 1:**
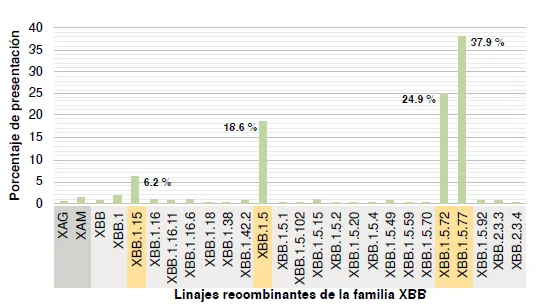
Distribución de linajes recombinantes de SARS-CoV-2 reportados entre el 9 de noviembre del 2022 y 5 de noviembre del 2023. En la franja gris claro se encuentran los linajes recombinantes más prevalentes que están representados en la familia XBB los cuales representan el 98,5 % de los genomas secuenciados.

Se caracterizaron 26 sublinajes recombinantes de ómicron a partir del análisis de los metadatos de 338 genomas de muestras positivas del departamento de Antioquia descargados de la base de datos GISAID. Se identificaron las subfamilias XAG, XAM y XBB; esta última presentó el mayor número de genomas recombinantes secuenciados (98,5 %) entre el periodo del 9 de noviembre del 2022 al 5 de noviembre del 2023, mostrando representatividad en 24 de 26 sublinajes recombinantes identificados. Los sublinajes de XBB más prevalentes fueron: XBB.1.5.77 (37,9 %; n = 128), XBB.1.5.72 (24,9 %; n = 84), XBB.1.5 (18,6 %; n = 63) y XBB.1.15 (6,2 %; n = 21) (figura 1).

**Figura 2:**
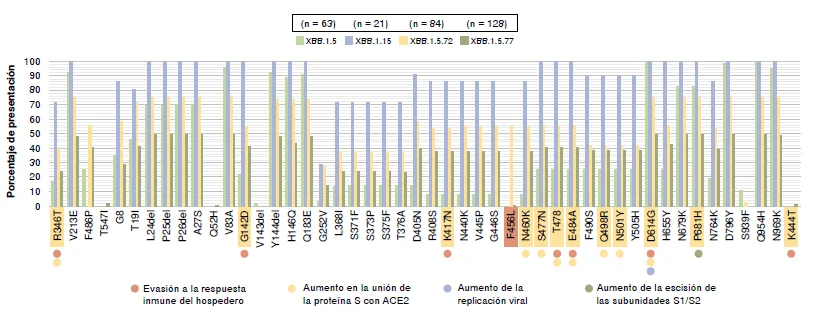
Representatividad de las mutaciones características de los linajes recombinantes XBB de mayor prevalencia (XBB.1.5, XBB.1.15, XBB.1.5.72 y XBB.1.5.77). En el recuadro amarillo están las doce mutaciones que han sido asociadas con evasión a la respuesta inmunitaria del huésped, aumento de unión de la proteína S con ACE2, aumento de la replicación viral y aumento de la escisión S1/S2.

El análisis de cambios de aminoácidos en los genomas de linajes recombinantes se restringió al gen S, dado que múltiples estudios han evidenciado el vínculo de algunas mutaciones identificadas en dicho gen con patogenicidad, infectividad, transmisibilidad o antigenicidad (cuadro 1) ^31^^,^^32^. Se caracterizaron 48 cambios de aminoácidos que han demostrado ser cambios puntuales para los cuatro linajes recombinantes más prevalentemente identificados en este estudio. De estos, se resaltan 12, los cuales han demostrado -según estudios previos- estar relacionados con eventos como: el aumento de la unión de la proteína viral S al receptor de la enzima convertidora de angiotensina 2 (ACE2, *angiotensin-converting enzyme* 2) de células huésped; el escape a la respuesta inmune del huésped; el incremento de la replicación viral y el aumento de la escisión de las subunidades S1/S2 (figura 2).

**Cuadro 1:** Mutaciones genéticas reportadas por previos estudios con evasión inmune y fusogenicidad

Cambio de aminoácido	Gen	Región	Función asociada	Linajes recombinantes	Porcentaje entre los linajes recombinantes
R346T	S	S1:RBD	Evasión de la respuesta inmunológica del huésped	XBB.1.5	17
				XBB.1.15	71
				XBB.1.5.72	40
				XBB.1.5.77	24
G142D	S	S1:NTD	Evasión de la respuesta inmunológica del huésped	XBB.1.5	22
				XBB.1.15	100
				XBB.1.5.72	55
				XBB.1.5.77	41
K417N	S	S1:RBD	Evasión de la respuesta inmunológica del huésped	XBB.1.5	8
				XBB.1.15	86
				XBB.1.5.72	54
				XBB.1.5.77	38
N460K	S	S1:RBD	Aumento de la unión a ACE2	XBB.1.5	8
				XBB.1.15	86
				XBB.1.5.72	55
				XBB.1.5.77	38
S477N	S	S1:RBD	Aumento de la unión a ACE2	XBB.1.5	25
				XBB.1.15	100
				XBB.1.5.72	56
				XBB.1.5.77	41
T478	S	S1:RBD	Aumento de la unión a ACE2 y escape de la respuesta inmunológica del huésped	XBB.1.5	25
				XBB.1.15	100
				XBB.1.5.72	56
				XBB.1.5.77	41
E484A	S	S1:RBD	Aumento en la unión a ACE2 y escape de la respuesta inmunológica del huésped	XBB.1.5	25
				XBB.1.15	100
				XBB.1.5.72	56
				XBB.1.5.77	41
Q498R	S	S1:RBD	Duplican el potencial electrostático y aumentan la afinidad de unión entre RBD y ACE2.	XBB.1.5	25
				XBB.1.15	90
				XBB.1.5.72	42
				XBB.1.5.77	38
N501Y	S	S1:RBD	Aumento en la unión a ACE2	XBB.1.5	25
				XBB.1.15	90
				XBB.1.5.72	42
				XBB.1.5.77	38
D614G	S	S1:SD2	Aumento de la unión a ACE2, escape de la respuesta inmunológica del huésped y mejora la replicación viral en las células epiteliales pulmonares humanas lo que aumenta la infectividad y la estabilidad de los viriones.	XBB.1.5	100
				XBB.1.15	100
				XBB.1.5.72	75
				XBB.1.5.77	49
P681H	S	S1:S1/S2	Aumenta levemente la escisión S1/S2	XBB.1.5	83
				XBB.1.15	100
				XBB.1.5.72	75
				XBB.1.5.77	49
K444T	S	S1:RBD	Evasión de la respuesta inmunológica del huésped	XBB.1.5	0
				XBB.1.15	0
				XBB.1.5.72	0
				XBB.1.5.77	1

ACE2: *angiotensin-converting enzyme 2*

La primera observación con respecto a estas mutaciones es que, en su gran mayoría, tienen una alta representatividad; para algunos sublinajes, como XBB.1.15, es de hasta el 100 %. Esto se explica porque el número de genomas obtenidos de este sublinaje durante el periodo epidemiológico evaluado fue de solo 21 genomas (cuadro 1, figura 2).

Para los cuatro sublinajes de ómicron más prevalentes (XBB.1.5, XBB.1.15, XBB.1.5.72 y XBB.1.5.77), se destacan las mutaciones D614G y P681H, las cuales muestran una representatividad de más del 48 %, llegando incluso a tener una presencia de hasta el 100 % para linajes como XBB.1.15. P681H es una mutación que ha evidenciado, por estudios previos, estar involucrada en el aumento de la escisión de las subunidades S1/S2, las cuales están estrechamente implicadas con la fusogenicidad de la membrana viral con la membrana de la célula huésped.

La mutación D614G está implicada en el aumento de la interacción entre la proteína S y el receptor de la ACE2, además de participar en el aumento de la replicación viral. Ambos eventos están vinculados con una mayor infectividad, así como pasa con P681H. Estas mutaciones pueden explicar la prevalencia de estos cuatro sublinajes durante el 2023. En la misma figura 2 se puede observar que el linaje más representativo entre los recombinantes es XBB.1.5.77 y que las mutaciones D614G y P681H, entre las mutaciones de importancia en los eventos infecciosos, siguen siendo muy representativas, alcanzando el 49 % de prevalencia en este linaje.

El resto de las mutaciones que no tienen un efecto comprobado sobre la transmisibilidad, virulencia o evasión a la respuesta inmunológica del huésped tienen una modesta representatividad; sin embargo, ninguna demuestra ser un biomarcador que permita discriminar entre los cuatro sublinajes de ómicron. XBB.1.5.77 es el único de los sublinajes de ómicron que tiene la mutación K444T en uno de sus genomas; no obstante, no es representativo de dicho sublinaje, dado que su presencia es de tan solo 0,008 % entre el grupo de genomas de XBB.1.5.77 analizados.

También se observó que el cambio F456L está presente en una alta frecuencia en XBB.1.5.72 (55 %) y, de manera infrecuente o ausente, en los demás sublinajes recombinantes. Este cambio de aminoácido demuestra ser un biomarcador que permite discriminar XBB.1.5.72 de otros sublinajes de XBB (figura 2).

### Análisis filogenético

**Figura 3:**
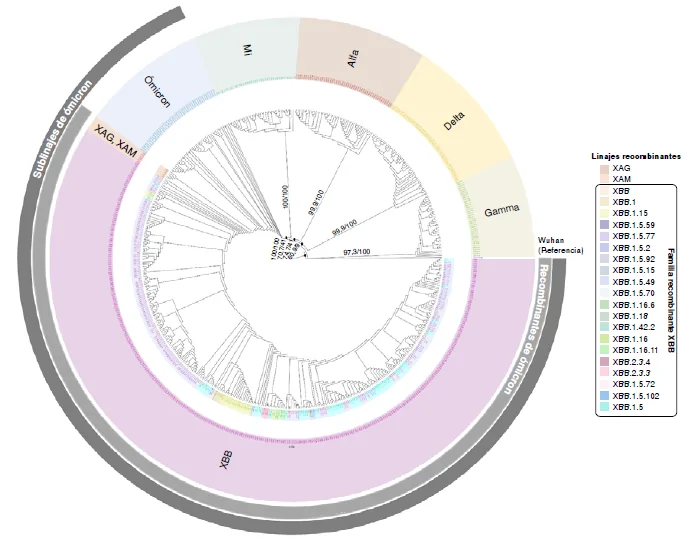
Árbol filogenético de linajes recombinantes de SARS-CoV-2 caracterizados entre el 9 de noviembre del 2022 y el 5 de noviembre del 2023. Se muestra la leyenda para cada uno de los principales ciados, así como para los linajes recombinantes XAG, XAM y XBB que forman parte de los sublinajes de ómicron. Se utilizó como genoma de referencia la secuencia Wuhan (NC_045512).

Los genomas de los diferentes linajes de SARS-CoV-2 analizados en este estudio se ubicaron en cinco grupos monofiléticos etiquetados como gamma, delta, alfa, mi y ómicron. Este último evidencia un grupo divergente etiquetado como «linajes recombinantes», los cuales comprenden tres familias: XAG, XAM y XBB (figura 3). En el árbol filogenético se observa que, entre el 9 de noviembre del 2022 al 5 de noviembre del 2023, en Antioquia, los linajes recombinantes más representativos pertenecían a la familia XBB (92,3 %). El árbol también permite evidenciar los 24 sublinajes de XBB, los cuales se muestran en diferentes gamas de colores, donde se hacen muy evidentes los dos más representativos, que son XBB.1.5.72 (24,9 %) y XBB.1.5.77 (37,9 %). En el caso de XBB.1.5.77, al principio de la rama se observa que forma el grupo divergente de ómicron (recombinantes), mientras que XBB.1.5.72 se ubica al final del árbol como el linaje recombinante de más reciente aparición entre este grupo rezagado.

### Análisis de recombinación e identificación de puntos de ruptura

Para este análisis se emplearon 15 genomas recombinantes, de los cuales cinco pertenecían a XBB.1.5, 5 a XBB.1.5.72 y 5 a XBB.1.5.77. La elección de estos tres sublinajes recombinantes se dio en función de su alta prevalencia durante el periodo epidemiológico evaluado en este estudio.

**Figura 4:**
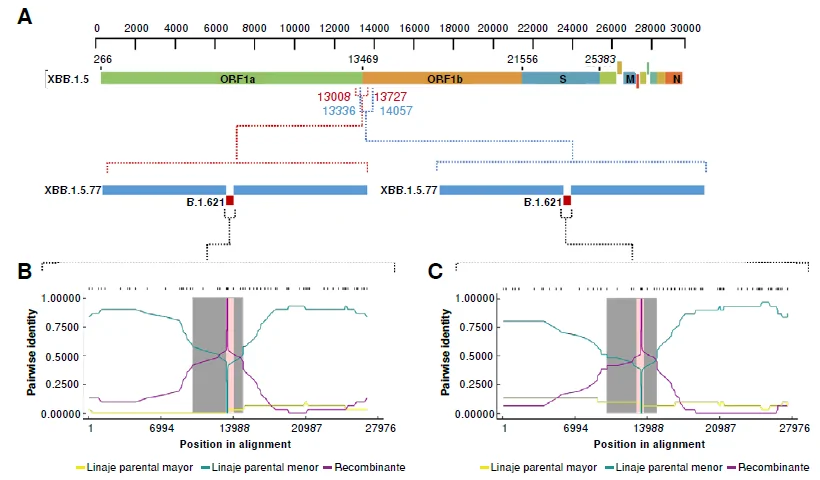
Análisis de recombinación mediante RDP, BootScan, MaxChi, Chimaera, 3SEQ, GeneConv, LARD y SiScan. **A)** La figura muestra dos sitios a lo largo del genoma del virus SARS-CoV-2, ubicados entre el gen *ORFIa* y *ORFIb,* que comprenden, para la figura de la derecha 719 pb y para la izquierda 721 pb. B-C) Los puntos de recombinación se pueden apreciar en la intersección de las líneas de diferentes colores, donde la línea morada representa el genoma recombinante y la línea amarilla y azul representan el linaje parental mayor y el menor, respectivamente. La interacción entre las líneas en la zona gris muestra la posición en el gen donde ocurre la recombinación. **B)** La figura muestra el punto de recombinación inicial en la posición 13008 y se extiende hasta el punto de recombinación final en 13727 y el fragmento que está involucrado en la recombinación es de 719 pb y se ve en la región resaltada en rosado que esta entre los genes *ORFIa* y *ORFIb.* **C)** La misma descripción se da para esta gráfica; sin embargo, los puntos de ruptura están entre 13336 y 14057 y el fragmento involucrado en la recombinación es de 721 pb. Estos son solo dos sitios de los tres que demuestran recombinación donde el que no se visualiza en la figura tiene puntos de ruptura en 13041 y 13727 donde el fragmento involucrado en el evento de recombinación es de 686 pb y ocurre entre el gen *ORFIa* y *ORFIb.* En A) se muestran dos ejemplos de dichos eventos de recombinación donde se evidencian como genomas recombinantes a XBB.1.5 y a los genomas parentales XBB.1.5.77 (linaje parental mayor) y B.1.621 (linaje parental menor). En la parte inferior de la figura se puede apreciar el mapa de recombinación donde las líneas de colores representan al genoma recombinante y a sus dos linajes parentales. Los puntos de cruce de dichas líneas evidencian el punto de recombinación que tiene dos lugares donde inicia el fragmento recombinado y donde termina el mismo.

La figura 4 muestra los eventos de recombinación genética que probablemente dieron origen a algunos de los genomas de la familia XBB. Como se refleja en el cuadro 2, de los 9 métodos que detectan genomas recombinantes solo 7 lograron encontrar genomas de este tipo. Métodos como Genecov, BootScan, MaxChi, Chimaera y 3Seq identificaron 10 genomas recombinantes, mientras que RDP encontró 8 y SiScan identificó un genoma de este tipo. LARD y PhyloLpro no lograron identificar genomas recombinantes (cuadro 2).

**Cuadro 2:** Métodos con sus respectivos p y número de genomas recombinantes identificados

Método	Número de genomas recombinantes detectados	p
RDP	8	1,881 x 10^-17^
Genecov	10	2,987 x 10^-16^
BootScan	10	7,691 x 10^-18^
MaxChi	10	1,063 x 10^-13^
Chimaera	10	2,047 x 10^-13^
SiScan	1	3,544 x 10^-21^
3Seq	10	1-771 x 10-^24^
LARD	---	---
PhyloLpro	---	---

El análisis con el gestor de métodos de identificación de puntos de recombinación RPD identificó como genomas recombinantes a XBB.1.5 y XBB.1.5.77 (cuadro 3). Teniendo en cuenta los resultados del RDP, siendo este el método más estricto de los nueve utilizados, se identificaron los puntos de recombinación de los linajes XBB.1.5 y XBB.1.5.77 (cuadro 3). En el caso del primero de estos genomas, XBB.1.5, RDP identificó dos muestras que eran producto de recombinación genética: hCoV-19/Colombia/ ANT-LDSP5029/2023 y hCoV-19/Colombia/ANT-LDSP5044/2023. En el primer caso, se identificaron los puntos de recombinación en la posición inicial 13008 (p = 1,584x10-9) en el gen *ORFIa* y en la posición final 13727 (p = 1,584x10-9) en el gen *ORFIb.* El segmento que se comprende entre estos dos puntos de ruptura alberga un fragmento 719 pb, cuyo linaje parental es B.1.621. Este es el linaje parental menor, dado que aporta en menor proporción al genoma recombinante hCoV-19/Colombia/ANT-LDSP5029/2023; en contraste, el linaje parental mayor es el genoma XBB.1.5.72, que es el que aporta el resto del genoma del recombinante.

En el caso del segundo genoma recombinante, hCoV-19/Colombia/ANT-LDSP5044/2023, se identificó el punto de ruptura inicial en la posición 13336 (p = 3,850x10-7) en el gen *ORFIa* y el punto de ruptura final en la posición 14057 (p = 2,735x10-17) en el gen *ORFIb.* Se identificó como genoma parental mayor a XBB.1.5.72 y como genoma parental menor a B.1.621.

Se identificaron seis de los genomas recombinantes de XBB.1.5.77, de los cuales tres tenían el punto de ruptura inicial en la posición 13041 en el gen *ORFIa* y el punto de ruptura final en la posición 13727 en el gen *ORFIb,* el cual comprende un fragmento de 686 pb cuyo linaje parental menor es B.1.621 y el linaje parental mayor es XBB.1.5.72 (cuadro 3).

**Cuadro 3:** Genomas XBB de mayor prevalencia entre el 9 de noviembre del 2022 y el 5 de noviembre del 2023, identificados como recombinantes

Recombinante	Linaje	Parental mayor	Parental menor	Punto de ruptura de inicio (p RDP)	Punto de ruptura de finalización (p RDP)	CÓDIGO GISAID
hCoV-19/Colombia/ANT-LDSP5044/2023	XBB.1.5	XBB.1.5.72	B.1.621	13336 (3,850 x 10^-07^)	14057 (2,735 x 10^-17^)	EPI_ISL_19046665
hCoV-19/Colombia/ANT-LDSP5029/2023	XBB.1.5	XBB.1.5.72	B.1.621	13008 (1,584 x 10^-19^)	13727 (1,584 x 10^-19^)	EPI_ISL_19046653
hCoV-19/Colombia/ANT-LDSP4888/2023	XBB.1.5.77	XBB.1.5.72	B.1.621	13041 (1,727 x 10^-23^)	13727 (1,727 x 10^-23^)	EPI_ISL_18160907
hCoV-19/Colombia/ANT-LDSP4878/2023	XBB.1.5.77	XBB.1.5.72	B.1.621	13041 (9,354 x 10^-15^)	13727 (9,354 x 10^-15^)	EPI_ISL_18160902
hCoV-19/Colombia/ANT-LDSP4812/2023	XBB.1.5.77	XBB.1.5.72	B.1.621	13008 (1,051 x 10^-17^)	13727 (1,051 x 10^-17^)	EPI_ISL_18160963
hCoV-19/Colombia/ANT-LDSP4878/2023	XBB.1.5.77	XBB.1.5.72	B.1.621	13041 (9,354 x 10^-15^)	13727 (9,354 x 10^-17^)	EPI_ISL_18160902
hCoV-19/Colombia/ANT-LDSP5048/2023	XBB.1.5.72	XBB.1.5.72	B.1.621	13336 (1,161 x 10^-08^)	14057 (1,750 x 10^-19^)	EPI_ISL_19046669
hCoV-19/Colombia/ANT-LDSP4857/2023	XBB.1.5.72	XBB.1.5.72	B.1.621	13336 (5,516 x 10^-08^)	14057 (1,576 x 10^-21^)	EPI_ISL_18160982

GISAID: Global Initiative on Sharing All Influenza Data

Otros dos genomas de este sublinaje de recombinantes tienen su punto de ruptura inicial en la posición 13336 el gen *ORF1a* y su punto de ruptura final en la posición 14057 en el gen *ORF1b,* el cual comprende un fragmento de 721 pb.

Finalmente, hay un genoma de XBB.1.5.77 que tiene un punto de ruptura inicial en la posición 13008 en el gen *ORF1a* y punto de ruptura final en la posición 13727 en el gen *ORF1b.* Todos los genomas recombinantes XBB.1.5.621 tienen como genomas parentales a XBB.1.5.72 (linaje parental mayor) y a B.1.621 (linaje parental menor) (cuadro 3).

## Discusión

La recombinación genómica se ha descrito ampliamente, siendo un fenómeno de vital importancia para la diversificación de los virus. La probabilidad de que esto suceda aumenta cuando diferentes variantes de un mismo virus circulan al mismo tiempo en el mismo lugar. En el caso del SARS-CoV-2, el primer recombinante reportado en el mundo fue XA en Reino Unido, bajo la nomenclatura de PANGO ^33^. Posteriormente, aparecieron variantes de este sublinaje, tales como XAG, XAM, XAY, XAZ. Esta última es altamente prevalente en Europa y Asia, mientras que XAM se encuentra casi exclusivamente en Norteamérica ^34^.

Una condición necesaria para la aparición del virus recombinante es la cocirculación, por lo menos, de dos linajes distintos del virus SARS-CoV-2, ya que la recombinación viral ocurre necesariamente durante eventos de coinfección en un único huésped ^35^^-^^37^. Esta condición se observó para los cuatro linajes más prevalentes de la familia XBB en Antioquia: XBB.1.5, XBB.1.15, XBB.1.5.72 y XBB.1.5.77, durante el periodo epidemiológico comprendido entre el 9 de noviembre del 2022 y el 5 de noviembre del 2023. El análisis descriptivo de los datos reveló distribuciones superpuestas de estos cuatro linajes, que evidenciaron cocirculación. Se pudo evidenciar que el linaje más representativo entre estos fue XBB.1.5.77 (37,9 %). XBB fue linaje recombinante que se extendió rápidamente por todo el mundo entre el 2022 y el 2023 ^38^.

Estudios previos, como el realizado en 2023 por Tamura *et al.*^39^, analizaron la aptitud viral de linajes de la familia XBB, indicando que este linaje recombinante es el primero de su tipo en demostrar un aumento sustancial con respecto al número de reproducción efectiva, a diferencia de otros linajes recombinantes tales como los de la familia XA y sus linajes parentales (BJ.1 y BM.1.1.1), lo que explica su rápido aumento entre la población humana ^38^. La investigación mencionada corresponde con los resultados de este estudio, en el que se identificaron cuatro linajes de XBB (XBB.1.5, XBB.1.15, XBB.1.5.72 y XBB.1.5.77) como los más prevalentes en la región de Antioquia. En la actualidad, XBB es la familia más representativa en las poblaciones del oriente y occidente del hemisferio y en las Américas ^39^^,^^40^.

En este estudio se caracterizó el perfil de cambios de aminoácidos que representa los cuatro principales sublinajes de XBB. Este perfil de mutaciones en los linajes recombinantes XBB demuestra la ventaja que dichos linajes tienen frente otras variantes del SARS-CoV-2, al comprender un perfil de mutaciones que le otorgan mayor eficacia de entrada, replicación y escape inmunológico.

Los perfiles mutacionales observados en los genomas recombinantes XBB revelan un enriquecimiento de cambios en la proteína de espícula *(spike)* que concuerdan con fenotipos de mayor transmisibilidad, evasión inmunitaria y eficiencia de entrada celular ^38^^,^^41^^-^^44^. Entre estas mutaciones se destacan D614G y P681H, presentes en los cuatro linajes principales, y N501Y que aparece en ~50 % de estos. D614G está vinculado con un aumento en la afinidad por ACE2 y la fusión membranal. Yurkovetskiy *et al.* demostraron que D614G mejora la exposición del RBD y eleva la entrada viral en células humanas, lo que explica su rápida fijación global ^45^. Otra mutación de importancia para los genomas recombinantes es P681H, la cual se ha asociado con el incremento de la eficiencia de clivaje y la fusogenicidad de la proteína de espícula. Lubinski *et al.* demostraron que P681H, al ubicarse adyacente al sitio de escisión, mejora la liberación de S1 y acelera la fusión de membranas, facilitando la entrada viral y contribuyendo al escape de interferones tipo I ^46^^,^^47^.

N501Y está vinculado con un aumento de la fuerza de unión a ACE2 ^48^. Simulaciones de Tian *et al.* (2021) evidenciaron que N501Y eleva la constante de afinidad del complejo RBD-ACE2 y aumenta las tasas de asociación ^48^^,^^49^, correlacionándose con una mayor tasa de transmisión observada en las variantes portadoras de esta mutación ^48^.

En conjunto, la coexistencia de estas mutaciones en los recombinantes XBB ubica a estos linajes en una posición competitiva superior frente a otros, al combinar mayor eficacia de entrada, replicación y escape inmune.

Este estudio identificó la mutación F456L únicamente en los linajes XBB.1.5.72 (55 %) y XBB.1.5.77 (1 %). Este cambio puede ser un biomarcador para discriminar estos dos sublinajes. Esto se evidencia en la revisión del 2023 de Focosi *et al.,* en la cual se identificó la mutación F456L en sublinajes recombinantes derivados de XBB ^50^.

Actualmente, en la base de datos outbreak.info se reporta esta mutación en los linajes XBB.1.5, XBB.1.5.72 y XBB.1.5.77, con una prevalencia de menos del 5 % en XBB.1.5 y en XBB.1.5.77, mientras que en XBB.1.5.72 se informa una prevalencia de cerca del 100 % ^51^. La mutación F456L ha demostrado proveerle ventajas al virus SARS-CoV-2: la primera es que se involucra en un aumento de la afinidad de la proteína S por ACE2; la segunda es que participa en la evasión de la respuesta inmunitaria al huésped, dado que estudios previos han demostrado que los linajes con F456L son menos sensibles a los anticuerpos neutralizantes del huésped ^50^.

En el análisis filogenético se evidenciaron cinco clados principales, conformados por alfa, delta, gamma, mi y ómicron. Vemos que ómicron es el último grupo derivado y que, además, evidencia ser un grupo divergente dentro de sus linajes, los cuales pertenecen, en su gran mayoría, a la gran familia XBB. El árbol muestra una clara división entre los sublinajes más prevalentes, XBB.1.5.72 y XBB.1.5.77. Este último es el primer sublinaje que se muestra en la rama del árbol del grupo de recombinantes, mientras que XBB.1.5.72 aparece en la última rama del mismo grupo divergente indicando que es el sublinaje más reciente en aparecer. La distribución de los *cluster* y los linajes recombinantes de ómicron se ajusta a lo que muestra la base de datos Nextclade y el estudio de 2024 de Wenhao Liu *et al.,* quienes analizaron las mutaciones en los diferentes sublinajes de ómicron asociadas con su evolución ^52^.

El análisis de los genomas recombinantes mediante RDP permitió identificar ocho genomas de este tipo: dos son XBB.1.5, cuatro son XBB.1.5.77 y dos son XBB.1.5.72. Para casi todos se identificó una pequeña región de 719 pb, comprendida entre la posición 13008 y 14047 y entre el gen *ORFIa* y *ORFIb.* En esta se da un evento de recombinación genética entre los linajes XBB.1.5.72 y B.1.621.

Cabe destacar que, aunque para estos ocho linajes recombinantes los linajes parentales resultan ser los mismos, sus diferencias radican en el perfil de mutaciones que albergan. Se resalta también que la detección precisa de los puntos de ruptura de recombinación depende del nivel de divergencia entre genomas de los diferentes linajes recombinantes analizados en este estudio, por lo que es de esperar que los puntos de ruptura cambien considerablemente en cada uno de los corridos independientes donde se analizaron los datos con el gestor de métodos RDP.

En nuestro caso, se tiene confianza en dichos puntos de ruptura dado que se hicieron tres corridos independientes donde los puntos de ruptura y las p de los métodos de recombinación no cambiaron entre corridos. Sin embargo, es de resaltar que la gran similitud genética y la corta divergencia entre los diferentes linajes de SARS-CoV-2 estudiados aquí pueden ser una variable para tener imprecisiones en las posiciones específicas de los puntos de ruptura ^52^.

El mapeo de estas regiones recombinantes proporciona una fuente de datos que permite evaluar la ocurrencia de recombinación genética a partir de los cambios de secuencia a nivel de los nucleótidos. Nuestros resultados demuestran coherencia con otros estudios, en los que se han identificado otros puntos de ruptura de recombinación, ubicados en la región comprendida entre el gen *ORFIa* y *ORFIb* y otros puntos de ruptura cerca al gen S descritos previamente para SARS-CoV-2 ^33^^,^^53^^-^^55^.

Aunque los registros de detección sugieren que B.1.621 desapareció el 25 de enero del 2022 y que XBB.1.5.72 surgió en 29 de abril del 2023, varios mecanismos explican cómo ambos linajes pudieron dar origen a los recombinantes XBB.1.5, XBB.1.5.77 y XBB.1.5.72 que se registran como parte de los resultados de nuestro artículo.

Una explicación del origen de los recombinantes XBB.1.5, XBB.1.5.77 y XBB.1.5.72 puede ser que los pacientes inmunocomprometidos sirvieron como reservorios de infecciones persistentes de SARS-CoV-2 por el linaje B.1.621 adquirido durante el periodo epidemiológico de septiembre del 2020 a enero del 2022. Estudios como el publicado en 2024 por Mahan Ghafari *et al.* demuestran que las infecciones persistentes por SARS-CoV-2 pueden actuar como reservorios virales que dan lugar a linajes altamente divergentes. Dicho estudio identificó, en 54 de 381 pacientes con COVID-19 persistente, que el ARN viral persistía al menos hasta 60 días. Esta investigación también estimó que la prevalencia de infecciones crónicas en la población es pequeña (0,1 al 0,5 %) y, por tanto, el virus puede pasar desapercibido para los sistemas de vigilancia de salud pública, lo que posibilita que la acumulación de mutaciones en su genoma y los eventos repetitivos de recombinación con otros linajes permitan que el virus regrese a la población general ^56^.

Otros estudios, como el publicado en 2020 por Choi *et al.,* reportaron un caso clínico de un paciente inmunocomprometido con infección persistente de SARS-CoV-2 durante 150 días. En ese periodo, el virus acumuló mutaciones, mostrando un microambiente que favorecía la generación de nuevas variantes dentro de un mismo huésped, lo cual provee un contexto idóneo para la coinfección y potencial recombinación ^57^.

Aunque los linajes como XBB.1.5.72 y B.1.621 tienen una superposición temporal de 459 días, que es la diferencia entre la fecha de desaparición del linaje B.1.621 y la aparición del linaje XBB.1.5.72, estudios como el de Jackson *et al,* publicado en el 2021, demuestran experimentalmente que un único episodio de coinfección puede dar origen a eventos de recombinación entre genomas virales de SARS-CoV-2, demostrando así que los episodios de coinfección aislados facilitan el intercambio de segmentos entre linajes ^9^.

También es de considerar que en los resultados obtenidos a partir de RDP4 se evidenció que en los genomas recombinantes de XBB.1.5, XBB.1.5.77 y XBB.1.5.72 la mayor parte de su genoma estaba conformado por el genoma del linaje XBB.1.5.72, mientras que una pequeña región de aproximadamente 700 pares de bases pertenecía al genoma del linaje B.1.621 ^9^. Esto sugiere que este estudio puede estar evidenciando un evento de recombinación más antiguo, el cual involucró al linaje B.1.621 con alguno de los linajes BA.2.10.1 o BA.2.75 que dieron origen a los linajes recombinantes del clado XBB de ómicron. Es probable que estos linajes comprendieran uno o varios fragmentos pequeños del genoma de B.1.621 que, posteriormente, en otros eventos de recombinación, fueran heredados a los genomas recombinantes XBB.1.5, XBB.1.5.77 y XBB.1.5.72. Estudios como el publicado en el 2021 por Jackson *et al.* han demostraron que los coronavirus, como el SARS-CoV-2, pueden recombinarse repetidamente a lo largo de su evolución y que los productos de esas recombinaciones pueden persistir en un linaje para luego actuar como donantes en eventos posteriores ^9^.

Una limitante inherente a nuestro estudio es la imposibilidad de realizar un seguimiento longitudinal a los pacientes inmunocomprometidos de nuestra cohorte para dar explicación al evento de recombinación entre las variantes XBB.1.5.72 y B.1.621 por persistencia de B.1.621 en pacientes con dicho fenotipo. En nuestro universo muestral, se registraron dos pacientes con linfoma B difuso de células grandes, un tipo de cáncer hematológico que compromete seriamente el sistema inmunitario. Estos pacientes se consideran inmunodeprimidos debido a la enfermedad subyacente y a los tratamientos sistémicos como la quimioterapia y el rituximab ^57^.

Investigaciones previas han documentado que los individuos inmunocomprometidos pueden albergar infecciones persistentes por SARS-CoV-2, sirviendo potencialmente como reservorios para la evolución viral y la generación de variantes ^58^^,^^59^. En nuestro análisis, esto podría haber permitido la persistencia del linaje B.1.621 en los pacientes identificados (EPI_ISL_18252291 y EPI_ISL_18252292), incluso después de la desaparición de este linaje en la población general durante el período de muestreo (septiembre del 2020 a enero del 2022). Dado que este estudio se basó en el análisis retrospectivo e *in silico* de datos de vigilancia, no fue posible confirmar si estos individuos desarrollaron una infección persistente, lo que constituye una limitación significativa.

La posible recombinación entre los linajes XBB.1.5.72 y B.1.621 es una hipótesis que debe ser evaluada cuidadosamente, especialmente en lo que respecta a la posibilidad de contaminación de muestras durante los procedimientos de laboratorio. Sin embargo, se descarta que la contaminación sea la explicación de este evento, dado que se implementaron rigurosos controles de calidad en cada etapa del flujo de trabajo, tanto en la preparación anterior a la PCR como después de ella.

Se utilizaron dos mezclas *(pools)* de cebadores compuestos por 218 oligonucleótidos prediseñados por el Instituto Nacional de Salud. Para mitigar el riesgo de contaminación cruzada, la preparación de la mezcla maestra pre-PCR se realizó en una cabina de bioseguridad clase II de tipo A2, de uso exclusivo para esta etapa. Además, por cada montaje de la PCR se incluyó un control negativo (solo mezcla maestra sin ADN molde), lo que nos permitió monitorear la presencia de cualquier contaminante.

Todos los procedimientos post-PCR, incluida la preparación de las librerías genómicas, se llevaron a cabo en un área física con equipos dedicados y separados de las áreas pre-PCR. El control negativo se mantuvo a lo largo de todo el proceso de preparación de la librería para verificar la ausencia de contaminación. Los análisis de cuantificación de las muestras, así como los análisis bioinformáticos subsiguientes, no mostraron evidencia de contaminación en los controles negativos, lo que descarta de manera concluyente la posibilidad de que la aparente recombinación sea el resultado de una contaminación cruzada en el laboratorio.
